# Ozone exposure upregulates the expression of host susceptibility protein TMPRSS2 to SARS-CoV-2

**DOI:** 10.1038/s41598-022-04906-8

**Published:** 2022-01-25

**Authors:** Thao Vo, Kshitiz Paudel, Ishita Choudhary, Sonika Patial, Yogesh Saini

**Affiliations:** Department of Comparative Biomedical Sciences, School of Veterinary Medicine, Louisiana State University, 1909 Skip Bertman Drive, Baton Rouge, LA 70803 USA

**Keywords:** Environmental impact, Acute inflammation

## Abstract

SARS-CoV-2, a novel coronavirus and an etiologic agent for the current global health emergency, causes acute infection of the respiratory tract leading to severe disease and significant mortality. Ever since the start of SARS-CoV-2, also known as the COVID-19 pandemic, countless uncertainties have been revolving around the pathogenesis and epidemiology of the SARS-CoV-2 infection. While air pollution has been shown to be strongly correlated to increased SARS-CoV-2 morbidity and mortality, whether environmental pollutants such as ground-level ozone affects the susceptibility of individuals to SARS-CoV-2 is not yet established. To investigate the impact of ozone inhalation on the expression levels of signatures associated with host susceptibility to SARS-CoV-2, we analyzed lung tissues collected from mice that were sub-chronically exposed to air or 0.8 ppm ozone for three weeks (4 h/night, 5 nights/week), and analyzed the expression of signatures associated with host susceptibility to SARS-CoV-2. SARS-CoV-2 entry into the host cells is dependent on the binding of the virus to the host cellular receptor, angiotensin-converting enzyme (ACE2), and its subsequent proteolytic priming by the host-derived protease, transmembrane protease serine 2 (TMPRSS2). The *Ace2* transcripts were significantly elevated in the parenchyma, but not in the extrapulmonary airways and alveolar macrophages, from ozone-exposed mice. The TMPRSS2 protein and *Tmprss2* transcripts were significantly elevated in the extrapulmonary airways, parenchyma, and alveolar macrophages from ozone-exposed mice. A significant proportion of additional known SARS-CoV-2 host susceptibility genes were upregulated in alveolar macrophages and parenchyma from ozone-exposed mice. Our data indicate that the unhealthy levels of ozone in the environment may predispose individuals to severe SARS-CoV-2 infection. Given the severity of this pandemic and the challenges associated with direct testing of host-environment interactions in clinical settings, we believe that this ozone exposure-based study informs the scientific community of the potentially detrimental effects of the ambient ozone levels in determining the host susceptibility to SARS-CoV-2.

## Introduction

SARS-CoV-2, a novel coronavirus and an etiologic agent of the current global health emergency, causes acute infection of the respiratory tract leading to severe disease and significant mortality^1^. SARS-CoV-2 entry into the host cells is dependent upon the binding of the viral spike (S) protein to the host cellular receptor, angiotensin converting enzyme (ACE2), and its proteolytic priming by the host-derived protease, transmembrane protease serine 2 (TMPRSS2)^2^. Therefore, the host susceptibility to SARS-CoV-2 could vary based on the expression of the host susceptibility proteins including ACE2 and TMPRSS2^3–5^. For example, increased expression of the host-derived protease, TMPRSS2, may promote the priming of SARS-CoV-2, thus resulting in increased infectivity and disease severity. The current literature indicates that individuals have varied susceptibility to SARS-CoV-2 that may be dependent on age^6,7^, gender^8^, underlying comorbidities^9^, and environmental pollution^10,11^. However, the list of factors determining varied susceptibilities of the human population to SARS-CoV-2 remains incomplete.

Nearly one-third of the United States population lives in areas with unhealthy levels of ozone^12,13^. While it is already known that the unhealthy levels of ozone increase the risk for developing cardiopulmonary health problems^14–20^, it is unclear whether the ambient ozone levels regulate the expression of host susceptibility proteins to SARS-CoV-2 and, in turn, account for the varied susceptibilities of the human population to SARS-CoV-2. Addressing this critical question is highly relevant in terms of increasing our mechanistic understanding of the host-air pollution (environment) interactions underlying the SARS-CoV-2 pathogenesis and epidemiology, and for developing future preventive and therapeutic strategies.

To begin to understand the impact of ozone inhalation on the host susceptibility to SARS-CoV-2, we analyzed lung tissues collected from mice that were sub-chronically exposed to filtered air or 0.8 ppm ozone for three weeks (4 h/night, 5 nights/week)^21^, and analyzed the expression of the gene and protein signatures associated with host susceptibility to SARS-CoV-2. We used western blotting and immunohistochemistry for assessing expression levels of TMPRSS2 protein in three different lung tissue compartments. To determine the RNA levels of *Tmprss2* and *Ace2* in a cell-specific manner, in situ gene expression was assessed for *Tmprss2* and *Ace2* genes using RNAscope approach. Finally, the mRNA expression levels of 33 known SARS-CoV-2 host susceptibility genes were assessed in three lung tissue compartments using RNASeq approach. Our findings indicate that host-environment interactions may modulate the expression of host susceptibility proteins to SARS-CoV-2 and prime the host to manifest severe respiratory illness following SARS-CoV-2 infection.

## Methods

### Animal husbandry, experimental design, and ozone exposure

Seven-week-old C57BL/6 J mice were procured from Jackson Laboratory (Bar Harbor, ME). Mice were maintained in individually ventilated, hot-washed cages on a 12-h dark/light cycle. Mice were housed in polycarbonate cages and fed a regular diet and water ad libitum. All animal experimentation procedures were approved by LSU Institutional Animal Care and Use Committee (IACUC) and performed in accordance with the ethical guidelines and regulations. The authors complied with the Animal Research: Reporting of In Vivo Experiments (ARRIVE) guidelines. Ozone was generated by an ozone generator (TSE Systems, Chesterfield, MO), and the ozone levels were monitored by UV photometric ozone analyzer (Envia Altech Environment, Geneva, IL). Data acquisition was done through DACO monitoring software (TSE Systems, Chesterfield, MO). Control mice were kept in a chamber supplied by filtered air (Air). Animals were exposed to ozone (800 ppb; 4 h/night, 5 nights/week, for 3 weeks) or air. Further details have been published previously^21^.

### Necropsy and tissue harvesting, micro-dissection of the extrapulmonary airways, the parenchyma, and purification of airspace macrophages

Animals were euthanized and tissues were collected for RNA isolation or histological analyses, as described previously^21^. Briefly, 12–16h after final exposure, the ozone- or air-exposed mice were anesthetized with an intraperitoneal injection of 2,2,2-tribromoethanol (250 mg/kg; Sigma-Aldrich, St Louis, MO). Midline laparotomy was performed to expose and severe inferior vena cava for exsanguination. Thereafter, thoracotomy was performed, and lungs were lavaged with a calculated volume (Body-weight in grams × 0.035 × 1000 = volume in µl) of ice-cold calcium- and magnesium-free Dulbecco’s phosphate-buffered saline (DPBS). Left lung lobes were formalin-fixed for immunohistochemical analyses. CD11b negative (CD11b−) BALF cells were harvested using CD11b-microbead kit, according to the manufacturer’s recommendations (Miltenyi Biotech, MA). Extrapulmonary airways and parenchymal regions of lungs were dissected, as described previously^21^.

### Immunohistochemistry for TMPRSS2

The immunohistochemical staining for TMPRSS2 was performed using previously published procedure^22–24^. Briefly, formalin-fixed, paraffin-embedded 5 µm lung sections were used for immunohistochemical localization of TMPRSS2. Sections were deparaffinized with Xylene and rehydrated with graded ethanol. Heat-induced antigen-retrieval was performed using a Citrate buffer (pH 6.0). Endogenous peroxidases were quenched with 3% hydrogen peroxide (10 min at room temperature). After blocking with 3% goat serum for 30 min, sections were incubated for 2h at room temperature with rabbit polyclonal TMPRSS2 primary antibody (ab214462; Abcam Cambridge, MA). The sections were then processed using VECTASTAIN Elite ABC HRP Kit (Vector Laboratories, Burlingame, CA), followed by chromogenic substrate conversion to insoluble colored precipitate using ImmPACT NovaRED HRP substrate Kit (Vector Laboratories, Burlingame, CA). Sections were counterstained with Gill’s Hematoxylin-I, dehydrated, and coverslipped with mounting media (H-5000, Vector Laboratories, Burlingame, CA).

### Western blotting for TMPRSS2

Whole lung homogenates aliquots were separated by SDS-PAGE (NuPAGE 4–12% Bis–Tris gradient gel; Life Technologies, CA) and transferred to the PVDF membrane. Rabbit polyclonal TMPRSS2 primary antibody (ab214462; Abcam Cambridge, MA) and mouse monoclonal alpha-tubulin (T5168, Sigma-Aldrich, MO) were used. Protein bands were visualized using secondary antibodies (Alexa fluor 680 Goat anti-rabbit IgG or Alexa fluor 800 Goat anti-mouse IgG) and acquired using Odyssey CLx, Imager (LI-COR, NE)^25^.

### In situ localization of *Tmprss2* mRNA

Formalin-fixed and paraffin-embedded 5 µm lung sections were used for in situ localization of *Tmprss2* mRNA using RNAscope technologies, as reported previously^21,23^. Briefly, formalin-fixed, paraffin-embedded 5 µm lung sections were deparaffinized with Xylene and rehydrated with graded ethanol. In situ hybridization was performed using Advanced Cell Diagnostics (ACD) proprietary RNAScope Technology (ACD, Newark, CA). Predesigned transcript-specific probes were used to hybridize *Tmprss2* and *Ace2* transcripts in the airway sections, and an RNAScope 2.5 HD Duplex Assay Kit (ACD) was used to amplify the transcript signals.

### RNA isolation and quality assessment, construction of sequencing library, RNA sequencing and gene expression analyses, and data availability

The detailed methodologies have been previously published^21^. The raw data have been submitted to the Gene Expression Omnibus (GEO) database. The data is available via https://www.ncbi.nlm.nih.gov/geo/query/acc.cgi?acc=GSE156799 . Heat maps for normalized gene expression values (Z-scores) were generated using GraphPad Prism 9.0 (GraphPad Software, La Jolla, CA).

### Statistical analyses

Student’s t-test was used to determine significant differences among groups. Statistical analyses were performed using GraphPad Prism 9.0 (GraphPad Software, La Jolla, CA). All data were expressed as mean ± standard error of the mean (SEM). A *p*-value < 0.05 was considered statistically significant.

## Results and discussion

TMPRSS2 is essential for the proteolytic priming of viral spike (S) protein of the SARS-CoV-2 following its binding to host receptor, ACE2. In fact, a recent study elegantly demonstrated that host cell entry of SARS-CoV-2 can be blocked by a clinically proven inhibitor of TMPRSS2 indicating the critical importance of TMPRSS2 in determining SARS-CoV-2 infectivity^2^. Therefore, the host susceptibility to SARS-CoV-2 could vary based on the expression of the host susceptibility proteins including ACE2 and TMPRSS2^3–5^. Individuals have varied susceptibility to SARS-CoV-2 that may be dependent on various factors including air pollution^10,11^. Air pollution levels correlate strongly with increased morbidity and mortality due to SARS-CoV-2^26–28^. While it is already known that the unhealthy levels of ozone, one of the 6 criteria air pollutants, increase the risk for developing cardiopulmonary health problems^14–20^, it is unclear whether the ambient ozone regulates the levels of expression of host susceptibility proteins to SARS-CoV-2 and in turn accounts, in part, for the varied susceptibilities of the human population to SARS-CoV-2. Therefore, we sought to test the hypothesis that ozone induces the expression of TMPRSS2 in lung tissue.

**Figure 1:**
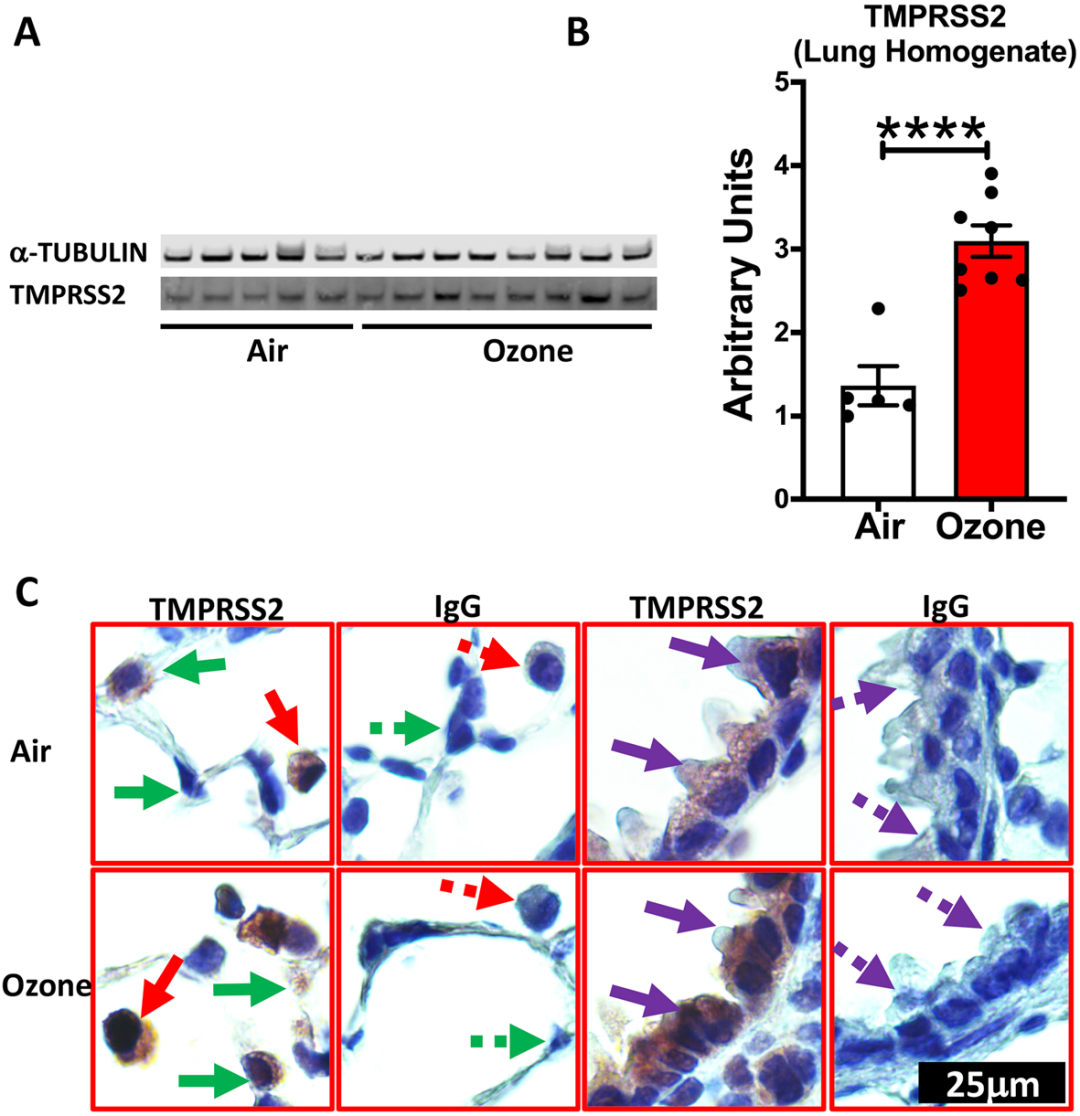
TMPRSS2 protein expression is upregulated in the lungs of ozone -exposed mice. Western blot representative gel image (**A**) showing bands for TMPRSS2 protein and alpha-tubulin loading control, and band intensity analyses (**B**, Bar Graph) on the whole lung homogenate from air- and ozone-exposed mice (n = 6–8). (**C**) Immunohistochemical staining for TMPRSS2 in macrophages (solid red arrow), alveolar epithelial cells (solid green arrow), and bronchiolar epithelial cells (solid purple arrow). Negatively stained cells are indicated by dotted arrows in lung sections that were incubated with antibody (IgG) control. All images were captured at the same magnification.

As compared to the filtered air-exposed mice, the ozone-exposed mice had significantly elevated levels of TMPRSS2 protein in the whole lung lysate (Fig. 1A and B; Supplemental Fig. 1). These results demonstrate that ozone exposure increases the expression of TMPRSS2 protein in the lungs of mice. Next, we performed immunohistochemical staining on lung sections to visualize cell-specific localization of TMPRSS2. Interestingly, while the TMPRSS2 staining was evident in the airway epithelium and alveolar macrophages from filtered air-exposed mice, the staining intensity was remarkably increased in the airway epithelial cells, alveolar epithelial cells, and alveolar macrophages from ozone-exposed mice (Fig. 1C). These results clearly demonstrate that ozone increases the expression of TMPRSS2 in the lung tissue in a cell-specific manner.

**Figure 2:**
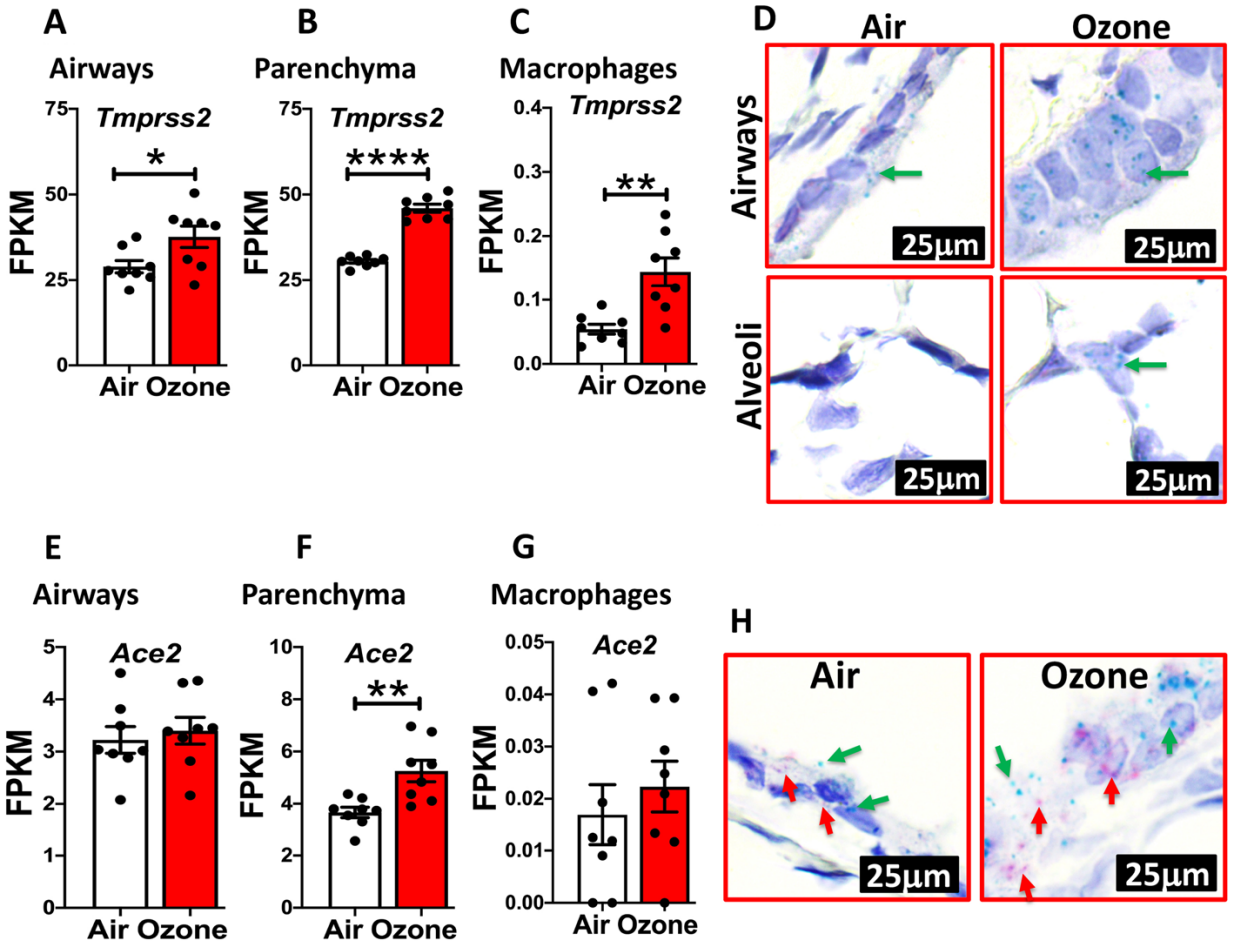
*Tmprss2* mRNA expression is upregulated in the lungs of ozone -exposed mice. FPKM values obtained from RNAseq data set obtained from airways (**A**), parenchyma (**B**), and alveolar macrophages (**C**) were used to quantify relative expression levels of *Tmprss2* mRNA in air- versus ozone-exposed mice (n = 8). The data are expressed as mean (± SEM). Student’s t-Test; *P < 0.05, **P < 0.01, ****P < 0.0001. (**D**) RNAscope-based in situ hybridization for *Tmprss2* transcripts (green dots representing punctate staining for *Tmprss2* mRNA in airway epithelial cells (Top) and alveolar epithelial cells (bottom) in air- (left) and ozone-exposed (right) mice. All images were captured at the same magnification. FPKM values obtained from RNAseq data set obtained from airways (**E**), parenchyma (**F**), and alveolar macrophages (**G**) were used to quantify relative expression levels of *Ace2* mRNA in air- versus ozone-exposed mice (n = 8). The data are expressed as means (± SEM). Student’s t-Test ** *P* < 0.01. (**H**) Duplex RNAscope-based in situ hybridization for *Tmprss2* (green puncta, green arrow) and *Ace2* (red puncta, red arrow) in the airways of air- (left) and ozone-exposed (right) mice. All images were captured at the same magnification.

In order to test whether TMPRSS2 protein expression correlates with mRNA expression in a cell-specific manner, we analyzed RNASeq dataset from extrapulmonary airways (Fig. 2A), parenchyma (Fig. 2B), and alveolar macrophages (Fig. 2C) from filtered air- and ozone-exposed mice for *Tmprss2* transcript levels. As expected, the fragment per kilobase per million mapped (FPKM) reads for *Tmprss2* were significantly upregulated in all the three tissue compartments in ozone-exposed as compared to filtered air-exposed mice. We further confirmed these findings for cell-specificity in ozone-exposed airways and alveoli using RNAscope-based in situ hybridization. This assay also showed significantly increased signals for *Tmprss2* transcripts in both the airway epithelial cells and the alveolar epithelial cells of ozone-exposed compared to filtered air-exposed mice (Fig. 2D). These data suggest that the changes in the protein levels of TMPRSS2 are a result of changes at the level of gene expression indicating that ozone directly or indirectly differentially regulates the gene expression of *Tmprss2*.

Next, to test whether ozone exposure also alters the expression of *Ace2* transcripts, we analyzed RNASeq dataset from extrapulmonary airways (Fig. 2E), parenchyma (Fig. 2F), and alveolar macrophages (Fig. 2G) from filtered air- and ozone-exposed mice for *Ace2* transcript levels. While the FPKM reads for *Ace2* were significantly upregulated in the parenchyma compartment (Fig. 2F) of ozone-exposed mice, the expression levels of *Ace2* in ozone-exposed extrapulmonary airways (Fig. 2E) and macrophage compartments (Fig. 2G) were not significantly different from filtered air-exposed mice. In situ hybridization revealed increased staining for *Ace2* transcripts in the airways of ozone-exposed mice (Fig. 2H). These data suggest that the ozone exposure results in a parallel increase in the expression of the two most critical SARS-CoV-2 host susceptibility genes.

**Figure 3:**
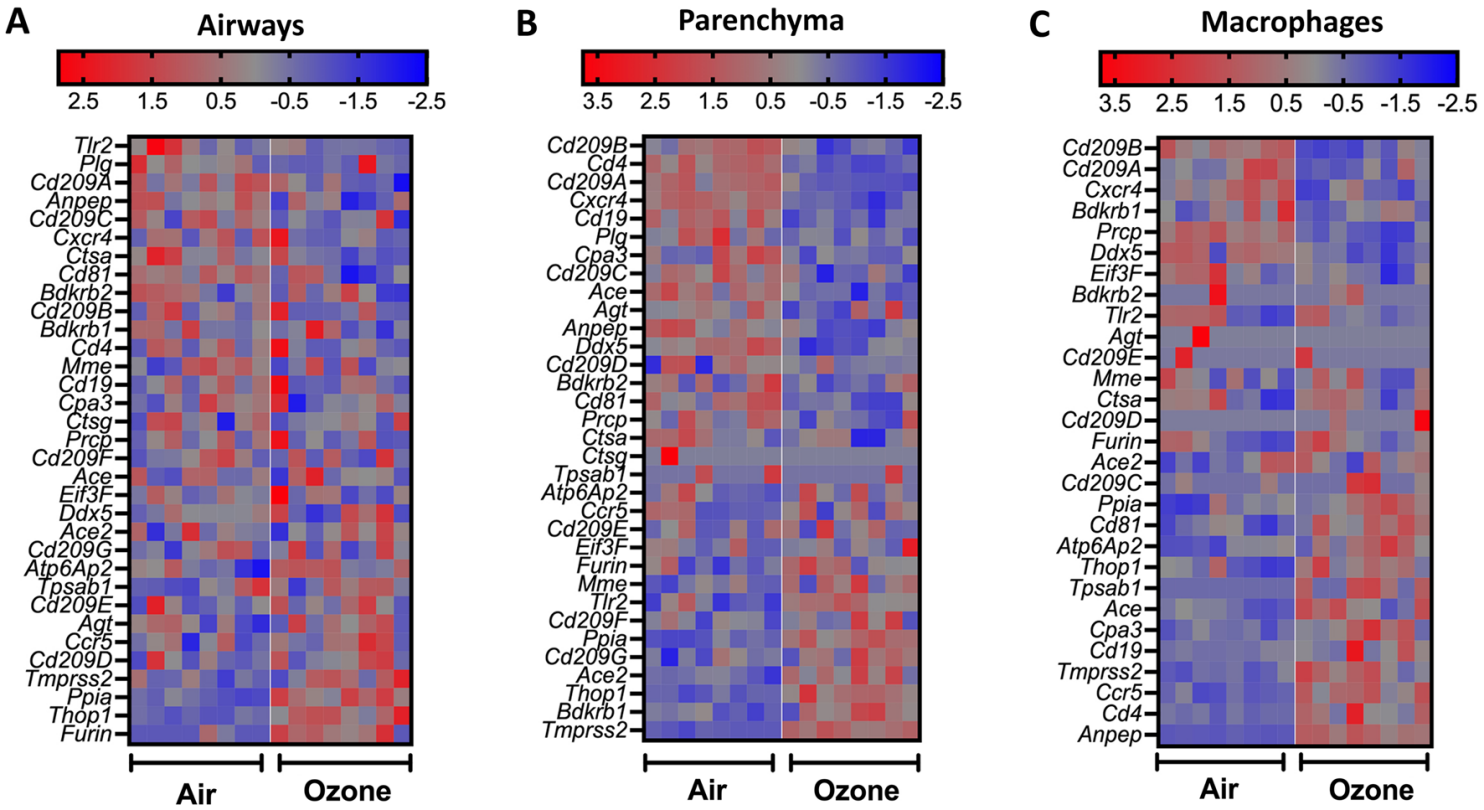
Heat maps depicting normalized gene expression values (Z-scores) of genes associated with SARS-CoV-2 host susceptibility in airways (**A**), parenchyma (**B**), and alveolar macrophages (**C**) from air- and ozone-exposed mice.

Finally, to determine the effect of ozone exposure on the expression levels of genes known to be involved in the host response to SARS-CoV-2, we compared the normalized z-scores of 33 known genes associated with host susceptibility to SARS-CoV-2^29–37^. Of the three tissues analyzed, while only four of the total 33 genes, i.e., *Furin, Thop1, Ppia, and Tmprss2*, were significantly increased in the extrapulmonary airways of ozone-exposed versus filtered air-exposed mice (Fig. 3A); in the lung parenchyma, nearly half of the host susceptibility genes were upregulated while the rest half were downregulated in ozone-exposed versus filtered air-exposed mice (Fig. 3B). In contrast to the extrapulmonary airways and the lung parenchyma, a major proportion of the host susceptibility genes including those of *Tmprss2, Ace, Anpep, Cd4,* and *Ccr5* were significantly upregulated in the CD11b- lung macrophages from ozone-exposed versus filtered air-exposed mice (Fig. 3C) indicating a large effect of ozone exposure on SARS-CoV-2 host susceptibility genes in CD11b− lung macrophages.

In terms of cell-specific responses, our data clearly indicate the upregulated expression of *Tmprss2* in alveolar macrophages. In addition to the alveolar macrophages, the interstitial macrophages may also exhibit similar expression trends for *Tmprss2* and other host susceptibility genes. While the interstitial macrophages are anatomically restricted to the interstitial spaces, the recently recruited inflammatory macrophages in SARS-CoV-2-infected rhesus macaques have been shown to exhibit interstitial cell-specific surface marker expression^38^. In addition, ozone exposure also induces the recruitment of CD11b + exudative macrophages into the lungs of mice^39^. In our study, since we selectively depleted CD11b + immune cells, the data presented in the current study reflect upregulated expression of *Tmprss2* and other host susceptibility genes specifically in CD11b− macrophages. It is quite possible that these inflammatory CD11b + macrophages may have even more exaggerated upregulation of *Tmprss2* and other host susceptibility genes. Within the parenchyma, in addition to the retained alveolar macrophages, a variety of immune cell types including myeloid cells such as neutrophils, eosinophils, classical dendritic cells and basophils, and adaptive immune cells such as B and T lymphocytes, and innate lymphoid cells such as ILC2 might have also contributed to the overall increase in the expression of *Tmprss2* and other host susceptibility genes including *Ace2*. For instance, CD4 + T cells, but not CD8 + T cells are also known to express *Tmprss2* and *Ace2* and these two host susceptibility proteins act in concert with CD4 to mediate SARS-CoV-2 infection^40^. Whether ozone exposure upregulates expression of *Tmprss2* and other host susceptibility genes in immune cells other than alveolar macrophages remains unexplored and requires further cell-specific transcriptomic and proteomic studies.

This study also has some limitations. First, mice are not susceptible to SARS-CoV-2 infection because SARS-CoV-2 is incapable of using murine ortholog of ACE2 for entering the host cells^41,42^. Therefore, we could not directly test the effect of ozone exposure on SARS-CoV-2 infectivity in mice. Future studies focused on the effect of ozone exposure on humanized ACE2 (hACE2) mice are essential. Second, the effect of ozone exposure on the expression of host susceptibility proteins in aged mice remains unexplored. Since elderly and older adults are high-risk patients for developing severe SARS-CoV-2 infection^43,44^, further investigations on three-dimensional relationship between ozone pollution, old age, and SARS-CoV-2 infectivity are warranted.

In conclusion, this study suggests a possible role of the host-environment (ozone pollution) interactions in modulating the susceptibility of the human population to SARS-CoV-2. Since TMPRSS2 is essential for the proteolytic processing of several coronaviruses including SARS-CoV-1, MERS, as well as influenza A virus^45,46^, our findings have implications beyond SARS-CoV-2 infections. Taken together, this study presents a novel finding that will have a significant and immediate impact on our understanding of the pathogenesis and epidemiology of the SARS-CoV-2 pandemic.

## Supplementary Information

Supplementary Information.
