# Supplementary material for: Ozone exposure upregulates the expression of host susceptibility protein TMPRSS2 to SARS-CoV-2
Source: Sci Rep. 2022 Jan 25;12:1357. doi: 10.1038/s41598-022-04906-8 (PMC8789794; doi:10.1038/s41598-022-04906-8)
Supplement: Supplementary file 1 — Supplementary Information. [file 41598_2022_4906_MOESM1_ESM.pdf]

Supplementary Figure 1. Western blot gel image

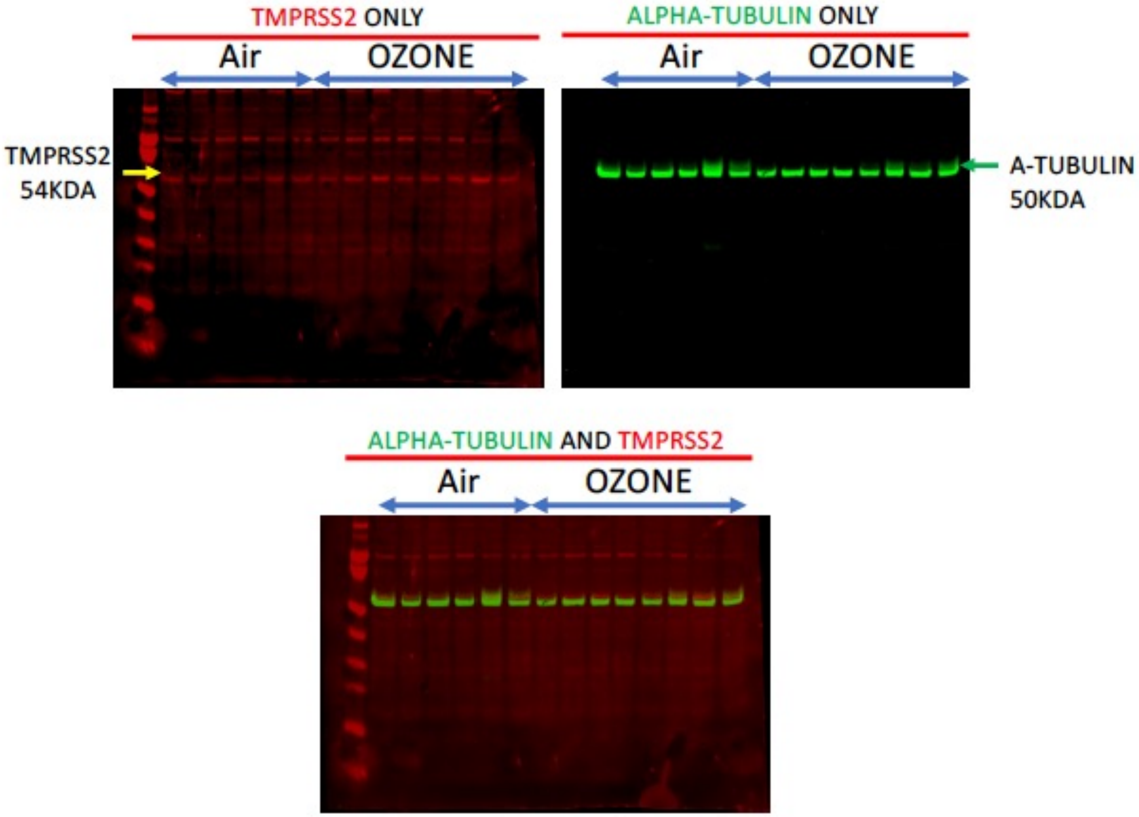

Supplementary Figure 1. Western blot gel image showing bands for TMPRSS2 protein on the whole lung homogenate from Air- and Ozone-exposed mice (n=6-8).
